# A preclinical PET dual-tracer imaging protocol for ER and HER2 phenotyping in breast cancer xenografts

**DOI:** 10.1186/s13550-020-00656-8

**Published:** 2020-07-03

**Authors:** Michel Paquette, Serge Phoenix, Christine Lawson, Brigitte Guérin, Roger Lecomte, Lee-Hwa Tai, Éric E. Turcotte, Jeffrey V. Leyton

**Affiliations:** 1 Department of Nuclear Medicine and Radiobiology, Université de Sherbrooke, 3001, 12e Avenue Nord, Sherbrooke (Qc), J1H 5N4 Canada; 2 Department of Anatomy and Cell Biology, Université de Sherbrooke, Québec, Canada; 3 Sherbrooke Molecular Imaging Center, Université de Sherbrooke, Québec, Canada; 4 Sherbrooke Pharmacology Institute, Faculty of Medicine and Health Sciences, Université de Sherbrooke, Québec, Canada

**Keywords:** Estrogen receptor, HER2, PET imaging, 4FMFES, [^89^Zr]Zr-DFO-Trastuzumab, Breast cancer

## Abstract

**Background:**

Nuclear medicine is on the constant search of precision radiopharmaceutical approaches to improve patient management. Although discordant expression of the estrogen receptor (ER) and the human epidermal growth factor receptor 2 (HER2) in breast cancer is a known dilemma for appropriate patient management, traditional tumor sampling is often difficult or impractical. While 2-deoxy-2[^18^F]fluoro-D-glucose (^18^F-FDG)-positron emission tomography (PET) is an option to detect subclinical metastases, it does not provide phenotype information. Radiolabeled antibodies are able to specifically target expressed cell surface receptors. However, their long circulating half-lives (days) require labeling with long-lived isotopes, such as ^89^Zr, in order to allow sufficient time for tracer clearance from the blood compartment and to accumulate adequately in target tumors and, thus, generate high-quality PET images. The aim of this study was to develop a dual-tracer PET imaging approach consisting of a fast-clearing small molecule and a slow-clearing antibody. This approach was evaluated in a model consisting of mice harboring separate breast cancer xenografts with either an ER+/HER2− or ER−/HER2+ phenotype, comparable to human metastatic disease with intertumor heterogeneity. Lastly, the aim of our study was to determine the feasibility of specifically identifying these two important phenotypes in an acceptable time window.

**Methods:**

Female nude mice were subcutaneously implanted on opposite shoulders with the ER+/HER2− and ER−/HER2+ MCF-7 and JIMT-1 tumor cell lines, respectively. A second model was developed consisting of mice implanted orthotopically with either MCF-7 or JIMT-1 cells. Pharmacokinetic analysis, serial PET imaging, and biodistribution were first performed for [^89^Zr]Zr-DFO-trastuzumab (^89^Zr-T) up to 8 days post-injection (p.i.) in JIMT-1 bearing mice. Region-of-interest (ROI) and biodistribution-derived uptake (% injected-activity/gram of tissue [%IA/g]) values and tumor-to-background ratios were obtained. Results were compared in order to validate ROI and identify early time points that provided high contrast tumor images. For the dual-tracer approach, cohorts of tumor-bearing mice were then subjected to sequential tracer PET imaging. On day 1, mice were administered 4-fluoro-11β-methoxy-16α-[^18^F]-fluoroestradiol (4FMFES) which targets ER and imaged 45 min p.i. This was immediately followed by the injection of ^89^Zr-T. Mice were then imaged on day 3 or day 7. ROI analysis was performed, and uptake was calculated in tumors and selected healthy organs for all radiotracers. Quality of tumor targeting for all tracers was evaluated by tumor contrast visualization, tumor and normal tissue uptake, and tumor-to-background ratios.

**Results:**

^89^Zr-T provided sufficiently high tumor and low background uptake values that furnished high contrast tumor images by 48 h p.i. For the dual-tracer approach, 4FMFES provided tumor uptake values that were significantly increased in MCF-7 tumors. When ^89^Zr-T-PET was combined with ^18^F-4FMFES-PET, the entire dual-tracer sequential-imaging procedure provided specific high-quality contrast images of ER+/HER2− MCF-7 and ER−/HER2+ JIMT-1 tumors for 4FMFES and ^89^Zr-T, respectively, as short as 72 h from start to finish.

**Conclusions:**

This protocol can provide high contrast images of tumors expressing ER or HER2 within 3 days from injection of 4FMFES to final scan of ^89^Zr-T and, hence, provides a basis for future dual-tracer combinations that include antibodies.

## Introduction

Most individual breast tumors expressing the estrogen receptor (ER) are managed well by oncologists due to the availability of several developed hormonal therapies. Overexpression of the human epidermal growth factor receptor 2 (HER2) used to be associated with a poor prognosis before the advent of HER2-targeted therapies, which greatly improved patient management. However, half of breast cancers that overexpress HER2 also express ER, and these types of tumors represent an unresolved clinical challenge and a major cause of treatment failure and mortality [1]. Currently, combination chemotherapy with anti-HER2 therapy is considered the best option for first-line treatment with patients with ER+/HER2+ breast tumors [1]. This is due to HER2 overexpression being an independent adverse prognostic factor [2, 3]. In addition, clinical studies have shown a poorer outcome for patients treated with hormonal therapy for ER+/HER2+ relative to ER+/HER2− patients [1].

Breast cancer biopsies of local or distant recurrences have resulted in ER and HER2 expression discordant from the original primary tumor sample [4, 5]. More importantly, studies have reported shorter survival for those with discordance between the primary and the recurrent breast tumors [6–9]. As a result, studies have reported changes in the treatment of relapsed patients according to the ER or HER2 phenotype of the metastatic tumor [10, 11]. The American Society of Clinical Oncology recommends physicians to biopsy accessible metastases and to perform immunohistochemistry (IHC) for ER and HER2 [12]. Thus, the oncology community realizes the significance of ER and HER2 phenotype discordance and the need for sampling of metastases.

There are several potential explanations for ER and HER2 intertumoral heterogeneity. Technical variability and subjective scoring for IHC-based determination have been shown to limit reproducibility for determining ER and HER2 expression on tumor specimens [4]. Metastatic lesions are often not accessible for biopsy due to ethical and logistical reasons. In addition, there may be heterogeneity between distant metastases from which only a single metastatic lesion is biopsied. Nevertheless, when a biopsy of a distant metastasis is available, ER and HER2 status should be reassessed, and the results should be evaluated in connection to the phenotype status of the original tumor.

Another explanation is that breast tumors can have an ER/HER2 phenotype switch, mostly due to selective pressure from targeted therapy. This is often referred to as “conversion” in recurrent or metastatic tumors that have occurred after post-treatment relapse [7, 8, 13, 14]. Although conversions are observed in the clinic, it has been preclinical investigations that have provided molecular insight for the ER-to-HER2 switch. Mice bearing ER+/HER2− MCF-7 tumors and treated with fulvestrant showed that HER2 was overexpressed in fulvestrant-resistant tumors [15, 16]. The reason has been narrowed to crosstalk between ER and other HER2 family members where both receptors are able to activate one another. Further details can be found in the review by Osborne et al. [17]. Thus, an evaluation method such as non-invasive imaging is a potential alternative approach to effectively assess ER and HER2 status.

Positron emission tomography (PET) imaging has demonstrated the ability to provide a great benefit for individually assessing ER and HER2 status, which has improved patient management. The PET tracer 16α-[^18^F]fluoroestradiol (FES) targets ER breast tumors and can accurately predict endocrine therapy response [18, 19]. [^89^Zr]Zr-DFO-trastuzumab (^89^Zr-T) can detect unsuspected HER2+ metastases in patients with HER2− primary breast cancer [20]. Although it is recommended that measurement of both ER and HER2 be performed on recurrent lesions [21], a combined noninvasive molecular imaging approach that provides a practical method to image whole-body ER and HER2 status does not exist despite the need. The obvious limitation is that each radiotracer used as a sole diagnostic imaging companion will not be able to detect both the ER+/HER2− or ER−/HER2+ phenotype discordance during a single PET scan.

Our group previously developed the PET tracer 4-fluoro-11β-methoxy-16α-[^18^F]fluoroestradiol (4FMFES) to improve detection of ER+ breast tumors by improving metabolic resistance of the radiotracer FES. Mice bearing ER+ breast tumors had improved tumor uptake and tumor-to-background contrast by PET when injected with 4FMFES relative to FES [22, 23]. In a phase II clinical trial with ER^+^ breast cancer patients, 4FMFES achieved a lower nonspecific signal and superior tumor contrast than FES PET, resulting in improved diagnostic confidence and lower false-negative diagnoses [24].

In this study, we present an approach to intentionally integrate 4FMFES-PET and ^89^Zr-T-PET in sequence and demonstrate its ability to detect ER+/HER2− and ER−/HER2+ tumors, respectively, in a shortened time window. Specifically, the approach consists of front-end injection and PET imaging with the rapid clearing 4FMFES tracer and scanning 45 min post-injection (p.i.), and the back-end injection and PET imaging with the slower clearing ^89^Zr-T administered immediately after and scanning performed at selected time points. The overall approach was evaluated for its ability to provide high contrast and to differentiate the ER+/HER2− MCF-7 and ER−/HER2+ JIMT-1 human breast tumors in female nude mice and whether it could occur at time points shorter than the 6 days p.i. observed in the clinic for ^89^Zr-T.

## Materials and methods

### Cell culture and reagents

MCF-7 was obtained from ATCC. JIMT-1 cells were a generous gift from Dr. Heikki Joensuu (University of Helsinki) [25]. Cell lines were grown in DMEM media supplemented with 1% amphotericin B, 1% penicillin/streptomycin, and 10% FBS (reagents supplied by Wisent, Canada).

### Animal model

Mice were handled in accordance with our institution’s Ethics Committee for Animal Experiments guidelines. Tumor implantation was performed under anesthesia with a mixture of 1.5% isoflurane and 2 L/min oxygen flow on female athymic nude mice (Charles River Laboratories, Wilmington, MA, USA). For ^89^Zr-T imaging optimization and pharmacokinetic evaluation, 5 × 10^6^ JIMT-1 cells were implanted subcutaneously in mice for longitudinal PET imaging and for biodistribution at multiple time points. A cohort of mice (*n* = 4) was implanted subcutaneously with 5 × 10^6^ MCF7 and JIMT-1 cells on each shoulder. For orthotopic tumors, 5 × 10^6^ MCF-7 (*n* = 4) or JIMT-1(*n* = 5) cells were implanted in a thoracic mammary pad. At the time of first imaging sessions, tumor volumes were 60–100 mm^3^, and by the end of the imaging sequences, no tumor had a volume of > 310 mm^3^.

### Radiotracer preparation

4FMFES radiosynthesis, purification, and activity were performed as already described [24]. For ^89^Zr-T preparation, trastuzumab was obtained from the clinical pharmacy at the Sherbrooke Medical Center. Trastuzumab (10 mg) was diluted in 0.1 M Na_2_HCO_3_ (pH 9.0) and reacted with a 10-fold molar excess of p-isothiocyanatophenyldeferoxamine (p-SCN-DFO) active ester (Macrocyclics, USA). After 30 min, the reacted trastuzumab was placed into Amicon Ultra 0.5-mL centrifugal filter (50 kDa cut-off) tubes (Millipore-Sigma, Canada) and centrifuged and buffer exchanged with PBS (pH 7.0). ^89^Zr-oxalate was produced as per the method by Alnahwi et al. [26]. One hundred megabecquerel of ^89^Zr-oxalate was neutralized with 2 M Na_2_HCO_3_ (pH 9.0) slowly while stirring. When the pH reached ≥ 6.5, 1 mL of 1 M HEPES buffer (pH 7.2) was added to the reaction tube. DFO-conjugated trastuzumab (1 mg) was introduced into the ^89^Zr-oxalate solution and incubated for 30 min at room temperature. ^89^Zr-T was purified using centrifugal filter tubes.

4FMFES radiochemical purity was measured as previously described [27]. A sample of 1 μg of ^89^Zr-T was evaluated by SDS-PAGE (4–15% gradient polyacrylamide gel) followed by autoradiography (Additional File 1a). In addition, ^89^Zr-T radiochemical purity was measured by instant thin-layer chromatography with 0.1 M DTPA as eluant (Additional File 1).

### Image reconstruction and ROI analysis for evaluating ^89^Zr-T uptake in JIMT-1 tumors and selected normal tissues over time

PET imaging sessions were performed on a LabPET8/Triumph small animal PET platform (Trifoil, CA, USA). JIMT-1 tumor-bearing female nude mice (*n* = 4) were intravenously injected with 2.0 ± 0.4 MBq of ^89^Zr-T and then imaged at 24 h, 48 h, 72 h, 144 h, and 168 h p.i., with each imaging sessions lasting for 15 min. Acquisition data were reconstructed using 20 iterations of a 3D Maximum Likelihood Expectation Maximization algorithm implementing a physical description of the detectors in the system matrix. Tracer distribution on the PET images was analyzed using the AMIDE software. A cylindrical phantom (24.8 mL) containing 1.4 ± 0.5 MBq of ^89^Zr at day 0 (with the same phantom was re-measured each subsequent ^89^Zr-T imaging day) or 2.4 ± 0.5 MBq of ^18^F for 4FMFES scans (for use in later sections) was used to obtain a calibration factor for converting the radioactive counts per second into percent injected activity/gram (%IA/g). Regions-of-interests (ROIs) were drawn for tumors, liver, muscle, heart, and bone, which were readily visible, as previously described [28]. Radioactivity uptake in the knee joint and the heart was used to determine general uptake for the “bone” and “blood,” respectively.

### Blood sampling and biodistribution at multiple time points for evaluating ^89^Zr-T in JIMT-1 tumor-bearing mice and comparison to ROI analyses

JIMT-1 tumor-bearing mice were injected with 1.8 ± 0.4 MBq ^89^Zr-T. Mouse cohorts (*n* = 3/group) had blood samples taken at days 1, 2, 3, 5, 6, and 8 h p.i. to calculate blood clearance rate. Mice were also euthanized by CO_2_ asphyxiation under deep isoflurane anesthesia, and biodistribution performed at 24 h, 72 h, 144 h, and 168 h. Organs of interest and tumors were collected and counted in a Packard Cobra II gamma-counter (GMI, Ramsay, MN, USA) for 1 min per tube with a 15–1000 keV energy window, background corrected and converted into %IA/g. Bone uptake reflected mostly the bone of the femur shaft, but there were portions of the knee joint included as separation from the lower limb involved slicing through the knee joint.

### Dual-tracer injection PET imaging protocol

The imaging protocol commenced with the intravenous (i.v.) injection of tumor-bearing mice with 2.6 ± 0.3 MBq 4FMFES followed by PET scans 45 min p.i. After the scan mice were immediately administered i.v. 3.9 ± 0.4 MBq ^89^Zr-T (50 μg) then returned to their cages. PET acquisitions were performed at 48 h and 144 h p.i. of ^89^Zr-T for the subcutaneous model and at 144 h only for the orthotopic model. Acquisition scan times of 15 min were performed for 4FMFES and 15 min and 30 min for ^89^Zr-T at 48 h and 144 h p.i., respectively.

### Statistics

Tissue uptake and tumor-to-background ratios for each group were reported as mean ± standard deviation. A Shapiro-Wilk normality test was performed on every dataset, which were all above the probability threshold set a priori at *p* < 0.05. Significance testing between radiotracers was performed using a 1-way ANOVA with Tukey’s multiple comparisons test, with a probability threshold set at *p* < 0.05.

## Results

### Longitudinal ^89^Zr-T-PET

**Fig. 1:**
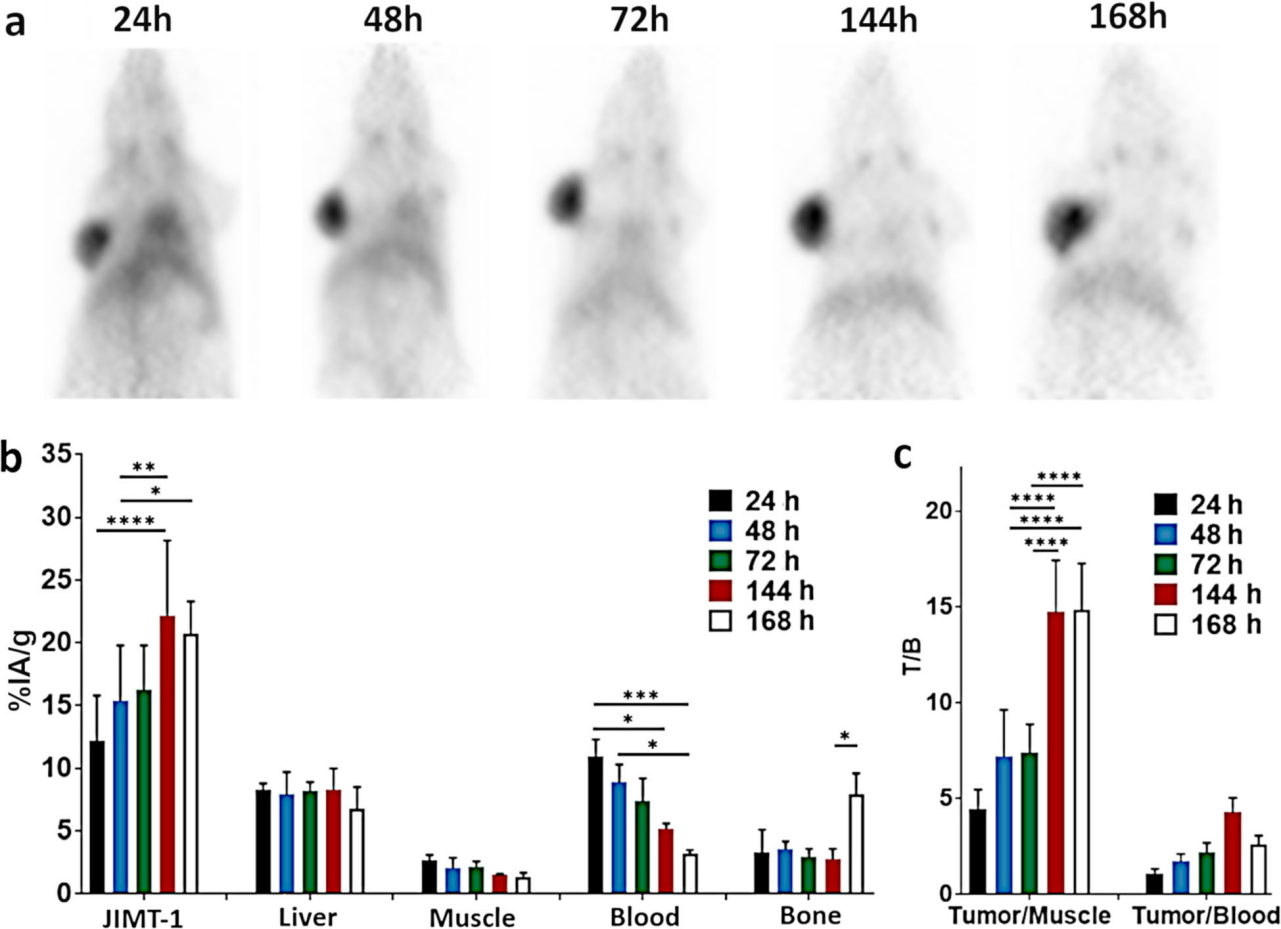
Longitudinal PET imaging following ^89^Zr-T injection in subcutaneously implanted HER2+/ER− JIMT-1 bearing mice. **a** Representative maximum intensity projection (MIP) images of the same mouse imaged at 24, 48, 72, 144, and 168 h post-injection of ^89^Zr-T. Images are all scaled at the same saturation level (0 to 15 %IA/g). **b** PET-derived ^89^Zr-T uptake in %IA/g of assessable tissues from 24 to 168 h post-injection. Blood uptake was estimated from signals originating from the heart cavity. **c** Tumor-to-muscle and tumor-to-blood ratios at all assessed time points. *p < 0.05; **p < 0.01; ***p < 0.005; ****p < 0.001

Longitudinal PET imaging of ^89^Zr-T injected (2.0 ± 0.4 MBq/mouse [*n* = 4]) in JIMT-1 bearing mice was performed to follow progression of the tumor uptake and to evaluate off-target accumulation over time. As shown in Fig. 1a, and as expected, image quality progressively improved over time. There was radioactivity present in the central cavity relative to radioactivity accumulated in tumors at 24 h. However, by 48 h, the radioactivity in the central cavity was noticeably reduced. The tumor uptake was strong in the tumor from 48 h to the final 168 h time point. As the background radioactivity reduced at 72 h and 144 h, the tumor contrast also proportionally increased. There was no visual difference in tumor contrast from 144 to 168 h. From a visual standpoint, sharp tumor images were evident at 48 h.

Tumor uptake increased steadily through most of the 168 h study. The tumor uptake at 48 h was 15.4 ± 4.4 %IA/g and increased to 16.3 ± 3.5 %IA/g at 72 h and peaked at 22.2 ± 6.0 %IA/g by 144 h (Fig. 1b). Radioactivity in the blood, as measured by ROI in the heart cavity, had a maximum uptake of 11.0 ± 1.3 %IA/g at 24 h and steadily decreased with values of 8.9 ± 1.4 %IA/g and 7.4 ± 1.8 %IA/g at 48 h and 72 h, respectively. The blood radioactivity decreased to a low of 3.2 ± 0.3 %IA/g by 168 h. The liver, muscle, and bone uptake remained steady and significantly lower than that of the tumor at all time points. Interestingly, a 2-fold uptake spike in the bone at 168 h was observed and most likely was caused by free ^89^Zr accumulation in the knee joint (Fig. 1b). The tumor uptake was significantly greater than the liver (*p* < 0.05), muscle (*p* < 0.001), and blood (*p* < 0.01) starting at the 48 h time point.

Tumor-to-muscle (T/M) ratios were ~ 7.5 at the early time points of 48 h and 72 h. The T/M ratios increased to ~ 15.0 at the later time points of 144 h and 168 h (Fig. 1c). In contrast, the tumor-to-blood (T/B) ratios started at 1.1 ± 0.3 at 24 h and increased steadily peaking to 4.3 ± 0.8 by 144 h (Fig. 1c).

Taken together, tumor uptake of approximately 15 %IA/g that resulted in T/M and T/B ratios of ~ 7.5 and 3.0, respectively, produced high contrast images of JIMT-1 tumors for ^89^Zr-T in a time window of 48–72 h p.i. As anticipated with intact antibodies, beyond 72 h, as tumor uptake increased and non-target uptake decreased, tumor contrast increased accordingly.

### ^89^Zr-T clearance and biodistribution and comparison to ROI analyses

**Fig. 2:**
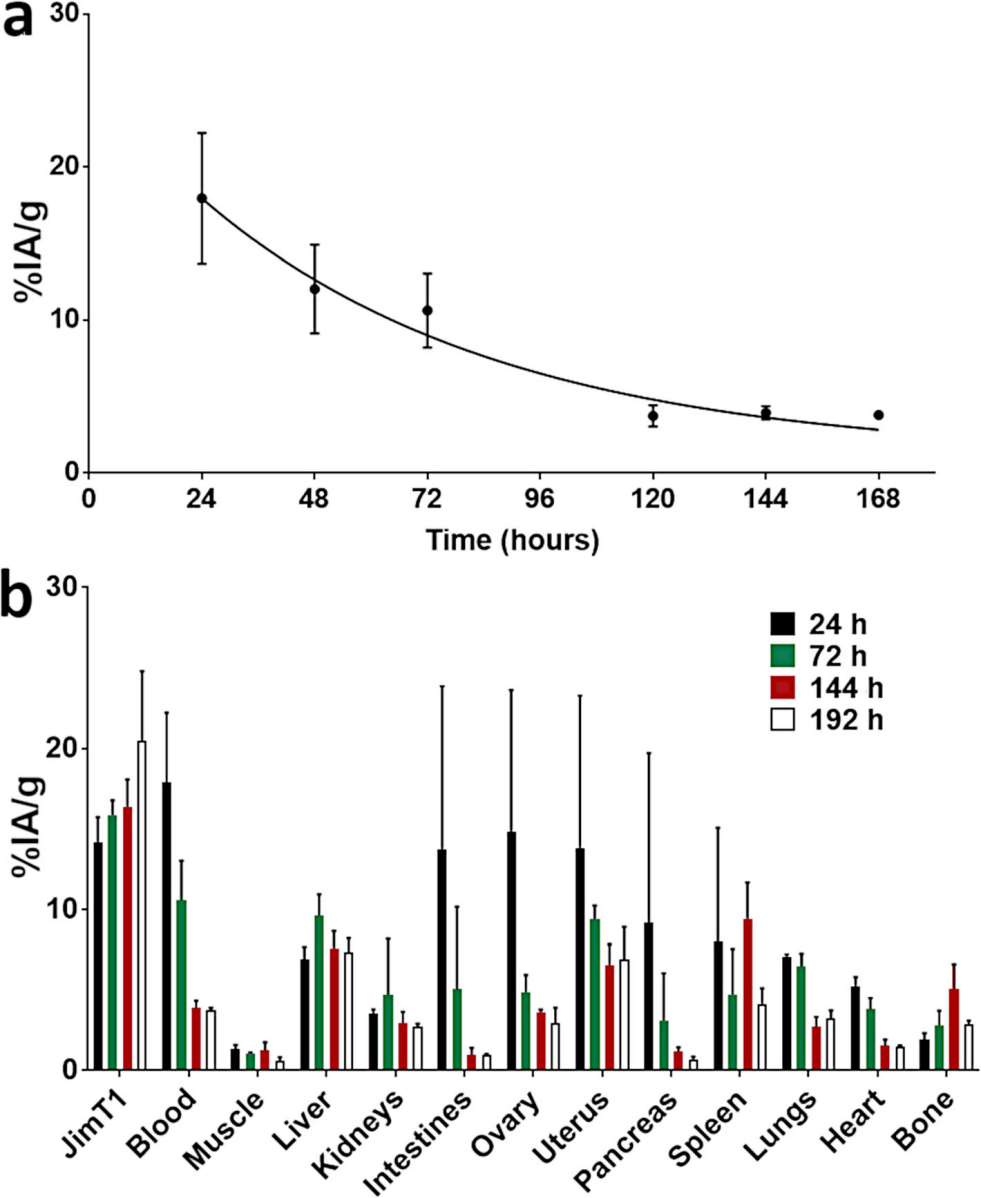
Pharmacokinetic assessment of ^89^Zr-T. **a** Blood curves derived from serial blood sampling. A mono-exponential fit, with formula *y* = 22.1e^−0.012x^ (*R*^2^ = 0.92) yielded a blood clearance half-life of 75 h. **b** Biodistribution profile at 24, 72, 144, and 168 h post-injection of ^89^Zr-T obtained from dissected organs. JIMT-1 tumors are the only tissue showing a continuous uptake increase through time, while most non-specific organs harbor either a decline or a stable uptake in the same time interval

In parallel to the mice previously analyzed by ROI, biodistribution was also performed on different tumor-bearing mouse cohorts. Physical blood sampling of ^89^Zr-T radioactivity in the blood gradually decreased according to an exponential fit with a half-life of 75 h (*R*^2^ = 0.92; Fig. 2a). The biological (whole-body residency) half-life, as determined by serial measurements of whole-body mice activity in a counting well compared to calculated radioactive decay of the injected activity following a linear regression (*R*^2^ = 0.91), was estimated at 234.4 h.

To determine whether the previous ROI measurements accurately reflected the circulating blood radioactivity, the heart was also dissected and contained radioactivity measured by gamma-counting. Uptake in the actual blood had a maximum uptake of 18.0 ± 4.7 %IA/g at 24 h and dropped to 12.0 ± 2.9 %IA/g and to 10.6 ± 2.4 %IA/g at 48 h and 72 h, respectively (Fig. 2b). At 120 h, the blood radioactivity fell nearly 3-fold to 3.7 ± 0.7 %IA/g and remained stable at time points 144 h, 168 h, and 192 h (Fig. 2a, b). Apart from the 24 h time point the ROI- and biodistribution-derived blood uptake values were comparable.

In contrast, the uptake in the heart derived from biodistribution did not match the uptake from the actual blood. The heart had a maximum uptake value of 5.3 ± 0.5 %IA/g at 24 h, which was markedly lower than the radioactivity in the actual blood at the same time point (Fig. 2b). By 144 h, uptake in the heart (1.6 ± 0.4 %IA/g) was decreased almost to background levels observed in the muscle (1.3 ± 0.5 %IA/g). Thus, the uptake in the blood, as measured on the heart by ROI, was in line, albeit with slightly reduced uptake values, with the radioactivity levels obtained from gamma counting on actual blood.

The biodistribution data of ^89^Zr-T revealed high and specific uptake in JIMT-1 tumors, which increased over time (Fig. 2b). JIMT-1 uptake was 14.2 ± 1.5 %IA/g at 24 h and increased to 15.9 ± 0.9 %IA/g and 16.4 ± 1.7 at 72 and 144 h, respectively. At 192 h p.i., the tumor accumulation was highest at 20.3 ± 4.3 %IA/g. Compared to ROI analysis, biodistribution showed that the tumor uptake curve was still rising through the final 192 h time point. In contrast, ROI showed that tumor uptake peaked at 144 h and then shouldered off at the final assessment time point of 168 h p.i. As with the ROI analysis, biodistribution also showed that tumor uptake was significantly increased compared to the liver (*p* < 0.05), muscle (*p* < 0.001), and blood (*p* < 0.05) starting at 72 h p.i.

Biodistribution-derived uptakes in the muscle, liver, and bone were very similar to the uptake values derived from ROI analysis. Interestingly, there was a spike in bone uptake from 72 h to 144 h and then dropped back down at 192 h. This pattern of bone uptake was discerned by both methods. Bone uptake at 168 h measured by ROI was 7.9 ± 1.7 %IA/g whereas by biodistribution uptake peaked to 5.1 ± 1.5 %IA/g at 144 h and then dropped to 2.9 ± 0.2 %IA/g at 192 h. This was most likely due to differences of bone sampling between both methods. Whereas ROI-derived uptake was determined by measuring radioactivity in the knee joint, biodistribution measured mostly the bone from the femur shaft. The knee joint which typically has the highest radiotracer content relative to the limb bones for ^89^Zr-T was most likely why the uptake values were higher by ROI analysis.

The T/M ratios calculated from biodistribution were 10.6 ± 1.6, 15.0 ± 0.9, 12.9 ± 3.2, and 32.6 ± 8.8 for 24 h, 72 h, 144 h, and 192 h, respectively. Although the T/M ratio was much lower by ROI analysis at 24 h (4.5 ± 1.0) (Fig. 1c), the ratios were comparable from 72 h and beyond. Importantly, at 72 h, the T/M by ROI of 7.5 matched closely with the T/M from the biodistribution data. The T/B ratios calculated from biodistribution were 0.8 ± 0.1, 1.5 ± 0.2, 4.2 ± 0.5, and 5.4 ± 0.7 for 24 h, 72 h, 144 h, and 192 h time points, respectively. This also matched well with the ROI-derived T/B ratios in Fig. 1c. The biodistribution-derived T/heart ratios were markedly increased at all time points relative to the T/B ratios, with values of 2.7 ± 0.7, 4.2 ± 0.8, 10.5 ± 1.7, and 13.4 ± 1.6 at 24 h, 72 h, 144 h, and 192 h, respectively. These results further support that the radioactivity measured by ROI in the heart region reflected the continual blood flow through this organ and not myocardium uptake.

The comparison between ROI- and biodistribution-derived uptake values and T/M and T/B ratios revealed that the two methods were comparable for determining ^89^Zr-T targeting of JIMT-1 tumors. However, there are slight discrepancies, but they can be explained by the differences between the two methodologies and further complicated by inter-group biological variations. Differences are most likely not caused by ^89^Zr-T synthesis as this radiotracer was repeatedly produced with high specific activity and purity. More importantly, these studies validated the use of ROI for moving forward and investigating the dual-tracer sequential-imaging approach and its ability to detect the ER+/HER2− and ER−/HER2+ phenotypes. In addition, this study identified that 48 h p.i. was suitable for ^89^Zr-T to produce high contrast tumor images, and we could integrate this time point into the dual-tracer protocol.

### Dual-tracer sequential-imaging protocol in mice bearing bilateral heterotopic tumors

**Fig. 3:**
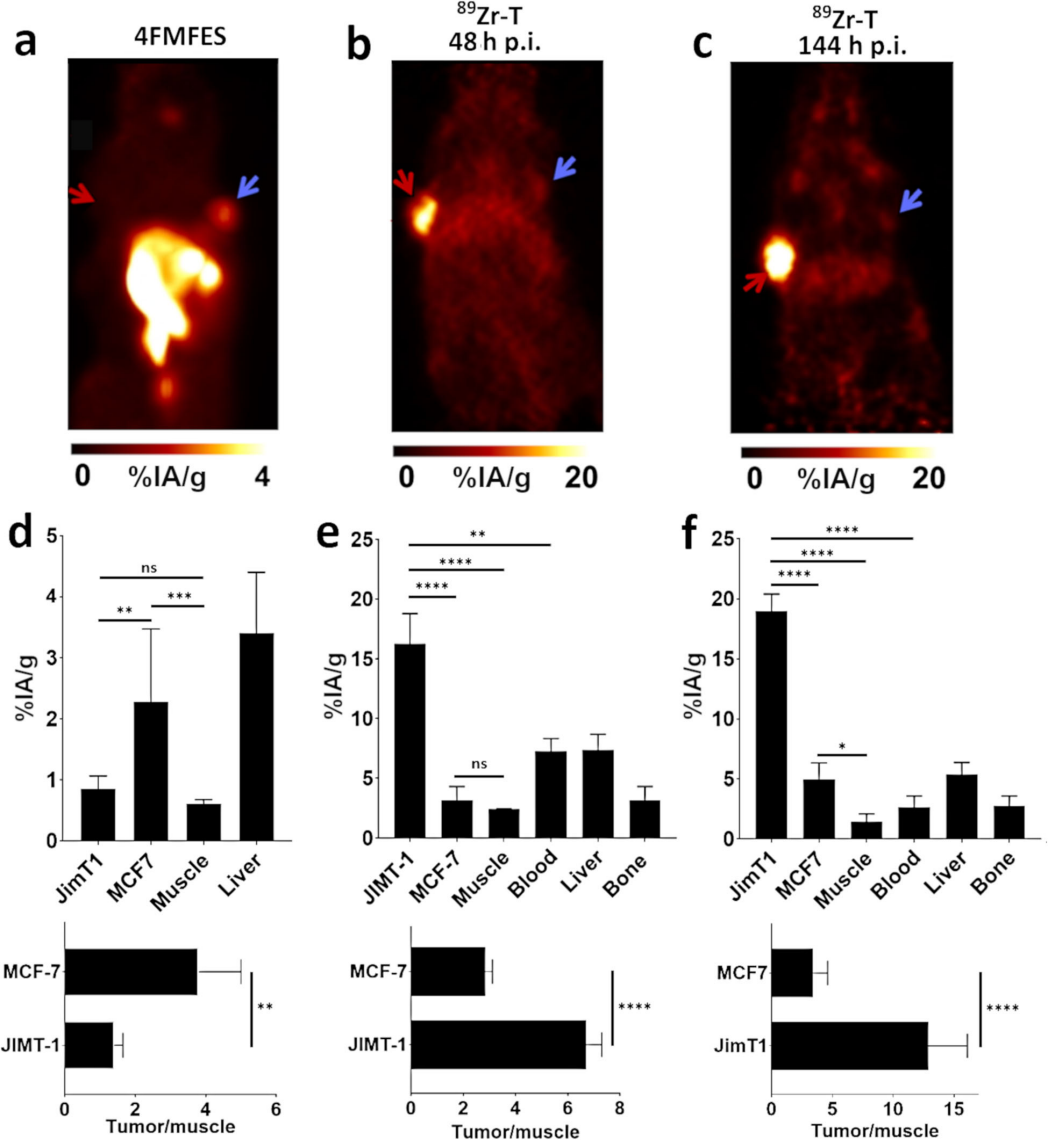
Sequential 4FMFES and ^89^Zr-T PET imaging on mice subcutaneously implanted with MCF-7 and JIMT-1 tumors. MIP image of a representative JIMT-1 (red arrows) and MCF-7 (blue arrows) of **a** tumor-bearing mice injected with 4FMFES or **b** with ^89^Zr-T and imaged at 48 h p.i. and **c** at 144 h p.i. PET-derived uptake of relevant organs in %IA/g (upper graphs) and tumor-to-muscle ratios (lower graphs) for **d** 4FMFES, **e**^89^Zr-T imaged at 48 h p.i., and **f**^89^Zr-T imaged at 144 h p.i. showing the phenotype-discerning capacity of the dual-tracer approach. *p < 0.05; **p < 0.01; ***p < 0.005; ****p < 0.001

Sequential injection and imaging by 4FMFES-PET followed by ^89^Zr-T-PET selectively discriminated ER+/HER2− MCF-7 and ER−/HER2+ JIMT-1 tumors, respectively (Fig. 3a). 4FMFES-PET detected the ER+/HER2− MCF-7 tumors but not ER−/HER2+ JIMT-1 tumors (Fig. 3a). By ROI analysis, the uptake value for MCF-7 tumors (2.3 %IA/g ± 1.2 %IA/g) was significantly (*p* < 0.005) increased over JIMT-1 tumors and muscle by factors of 2.6 and 4.6, respectively (Fig. 3d). Uptake in the intestines was observed and indicated normal hepatobiliary elimination for estradiol-based tracers in mice and humans [23, 24]. Accordingly, the liver uptake value was 4.0 %IA/g ± 0.6 %IA/g. The uptake in the MCF-7 xenografts but not JIMT-1 tumors was significantly (*p* < 0.005) increased over uptake in the muscle (Fig. 3d).

Immediately following 4FMFES-PET, mice were intravenously injected with ^89^Zr-T. Selecting the 48 h p.i. time point from Fig. 1a, ^89^Zr-T-PET revealed strong tumor signals in ER−/HER2+ JIMT-1 but not in ER+/HER2− MCF-7 tumors (Fig. 3b). The ROI-derived uptake value in the JIMT-1 tumors was 16.3 %IA/g ± 2.5 %IA/g, which was significantly (*p* < 0.001) increased over MCF-7 by a factor of 2.4 at 48 h (Fig. 3e). As anticipated, high levels of circulating ^89^Zr-T were observed but well below the JIMT-1 tumor PET signal intensities. As a result, JIMT-1 tumor uptake was also significantly (*p* < 0.001) increased relative to uptake in the muscle, liver, and blood. The late time point of 144 h revealed increased signals from JIMT-1 relative to the 48 h time point (Fig. 3c). The radioactivity from circulating ^89^Zr-T and in all tissues was relatively low, with the exception of the liver, which presented a discernable uptake. The uptake value in JIMT-1 tumors increased from 13.7 ± 1.9 %IA/g at 48 h to 19.0 ± 1.0 %IA/g at 144 h, whereas the uptake value in MCF-7 tumors decreased in the same time interval (5.9 ± 2.3 %IA/g to 5.0 ± 1.4 %IA/g) and could not be discerned in the image (Fig. 3f). ^89^Zr-T uptake in the muscle and liver remained stable. Radioactivity in the blood decreased from 7.3 ± 1.1 %IA/g to 2.7 ± 0.9 %IA/g for 48 h and 144 h, respectively. ^89^Zr-T achieved significantly increased (*p* < 0.001) uptake margins between JIMT-1 tumors and the MCF-7 tumors and healthy organs at the later time point. The most commonly used chelator for ^89^Zr is DFO; the ^89^Zr-DFO complex is partly unstable, and as a result, there can be substantial nonspecific accumulation in the bone in preclinical tumor models at later time points [29–31]. Nonetheless, the uptake in bone at 144 h was reasonably low at 5.1 ± 1.5 %IA/g compared to previously published reports (Fig. 3f). Importantly, uptake values in the tumor and organs for ^89^Zr-T matched well with the ROI-derived uptake values during the imaging optimization studies with ^89^Zr-T only (Fig. 1).

Comparing the analyzed T/background, radioactivity uptake ratios in tumors and healthy organs provided increased information on the feasibility of 4FMFES and ^89^Zr-T to specifically image the ER+/HER2− and ER−/HER2+ phenotypes, respectively. 4FMFES-PET revealed MCF-7 and JIMT-1 T/M ratios of 3.7 and 1.4, respectively. ^89^Zr-T-PET at 48 h p.i. revealed a JIMT-1 T/M ratio of 6.7, and T/liver and T/blood ratios were both 2.2. The JIMT-1 T/M, T/liver, and T/B ratios increased proportionally at 144 h to values of 8.5, 3.5, and 7.1, respectively, whereas the T/bone ratio reached 2.0 (Fig. 3f). The MCF-7 T/M and T/B ratios at 144 h were between 1.8 and 2.8, respectively, and significantly lower than those observed for JIMT-1 tumors (*p* < 0.001). Hence, the PET images, uptake values, and T/background ratios collectively demonstrated the feasibility of the dual-tracer protocol to provide ER+/HER2− and ER−/HER2+ tumor targeting specificity within a week and as early as 72 h from start to finish.

### Dual-tracer sequential-imaging protocol in mice bearing orthotopic tumors

To further evaluate the protocol capacity to discriminate between ER+/HER2− and ER−/HER2+ tumors with high contrast visualization in a more patient-reflective microenvironment, the location of the tumors was changed from heterotopic to orthotopic. In addition, mammary fat pads were chosen for tumor growth that were in close anatomical proximity to the hepatobiliary system, which is the major metabolic sink for 4FMFES and ^89^Zr-T.

**Fig. 4:**
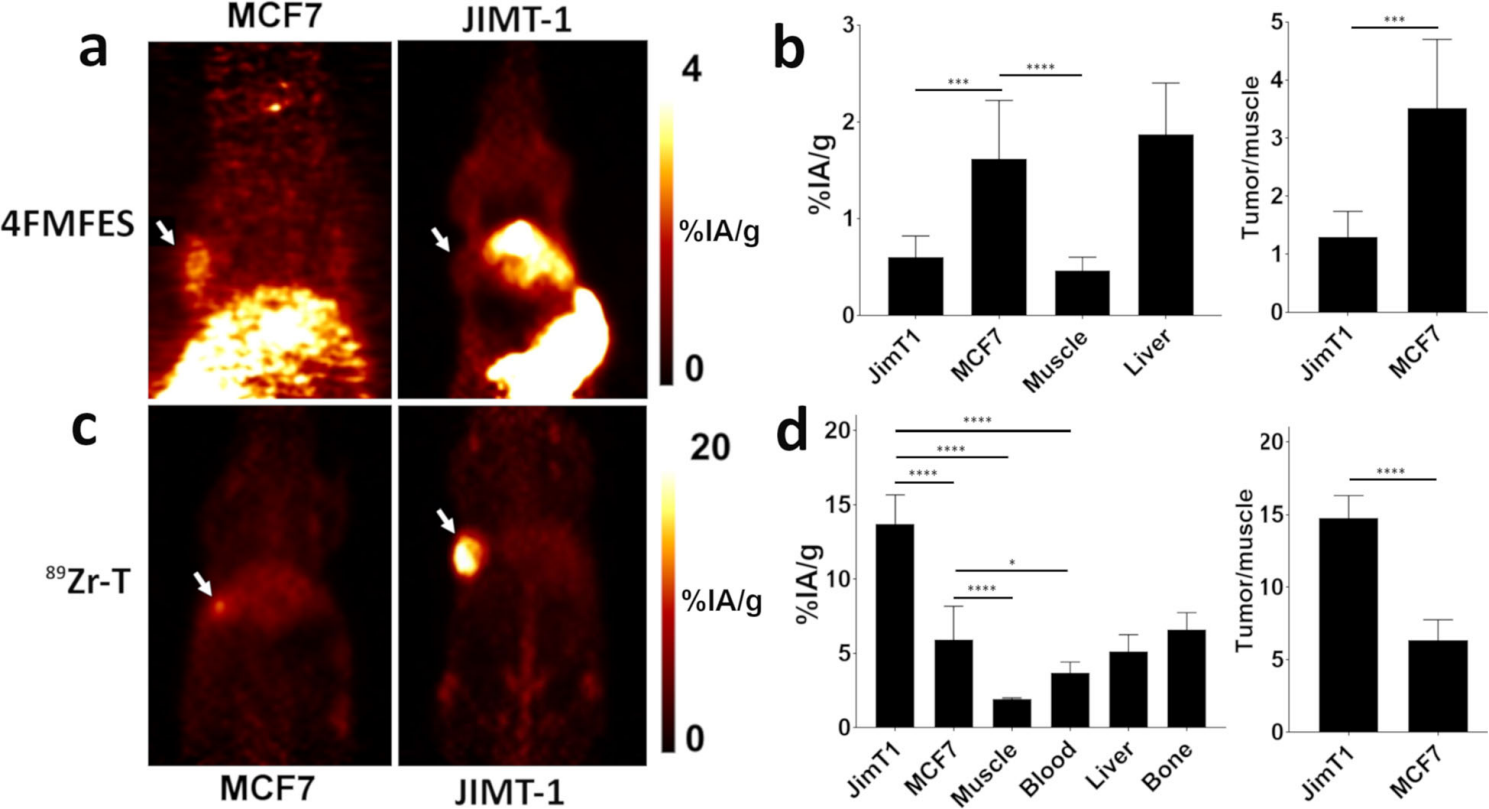
Sequential 4FMFES and ^89^Zr-T PET imaging in an orthotopic MCF-7 or JIMT-1 mouse model. **a** Representative coronal slices of 4FMFES-injected mice bearing either MCF-7 at 45 min p.i. or JIMT-1 tumors (white arrows). **b** 4FMFES PET-derived uptake reported in %IA/g of relevant organs and tumor-to-muscle ratios for each tumor type. **c** Representative coronal slices of ^89^Zr-T at 144 h p.i. of MCF-7 or JIMT-1 tumor-bearing mice (white arrows). **d**^89^Zr-T PET-derived uptake of relevant organs in %IA/g and tumor-to-muscle ratios for each tumor type. *p < 0.05; ***p < 0.005; ****p < 0.001

Mice injected with 4FMFES produced PET images where signals from the MCF-7 tumors were well above surrounding muscle tissue and could be discerned from the high background signals from the hepatobiliary system (Fig. 4a). In contrast, the JIMT-1 tumors produced a low background signal equivalent to the signals coming from the surrounding muscle tissue. The uptake value for MCF-7 tumors (1.6 ± 0.6 %IA/g) was significantly (*p* < 0.005) increased over JIMT-1 tumors (0.6 ± 0.2 %IA/g) (Fig. 4b). The uptake in the MCF-7 xenografts but not JIMT-1 tumors was significantly (*p* < 0.001) increased over uptake in the muscle.

At 144 h, ^89^Zr-T-PET showed strong PET signals from ER−/HER2+ JIMT-1 tumors with minimal background including the liver (Fig. 4c). Unlike the heterotopic model, PET images did show signals from ER+/HER2− MCF-7 tumors that had marginal increased intensity relative to signals from the liver, which was in close proximity. The uptake value in the JIMT-1 tumors was 13.7 ± 1.9 %IA/g, which was significantly (*p* < 0.001) increased over MCF-7 by a factor of 2.3 (Fig. 4e). The radioactivity from circulating ^89^Zr-T and in all tissues with the exception of the liver had scant radioactivity. ^89^Zr-T accumulation in JIMT-1 tumors was significantly increased relative to the MCF-7 tumor, liver, muscle, blood, and bone (Fig. 4d).

The tumor uptake values obtained for both radiotracers in the orthotopic model were slightly decreased compared to the uptake values from the heterotopic tumor model (Fig. 3a–c; Fig. 4a, b). ^18^F-4MFES uptake also decreased, albeit non-significantly (*p* = 0.35) in both tumors compared to the heterotopic tumor model. The uptake for ^89^Zr-T in target JIMT-1 tumors was reduced by ~ 4% at 6 days p.i. However, uptake in healthy tissues for both radiotracers was also reduced, most likely because of inter-group variability. Hence, the T/background ratios were similar between both models. For instance, MCF-7 T/M ratios for 4FMFES were 3.7 and 3.5 for the heterotopic and orthotopic models, respectively (Fig. 3d and Fig. 4b). JIMT-1 T/M ratios for ^89^Zr-T were 6.7 and 6.4 for heterotopic and orthotopic models, respectively (Fig. 3f and Fig. 4d). Thus, the PET images, uptake values, and T/background ratios in the orthotopic model were comparable to the results obtain in the heterotopic tumor model.

## Discussion

These results indicate that the targeted tracers ^18^F-4FMFES and ^89^Zr-T when administered and imaged in sequence could visually discriminate breast tumors with the ER+/HER2− and ER−/HER2+ phenotypes within a time period as short as 3 days. This was supported by data demonstrating excellent T/background ratios for both 4FMFES and ^89^Zr-T in their respective specific tumor targets. The non-negligible uptake in HER2− MCF-7 tumors is consistent with the known background that can cause false positives in HER2− patients for this radiotracer [32]. In addition, this work provides a preclinical guide for a dual-tracer approach that incorporates a fast-clearing small-molecule and slow-clearing intact antibody to determine the status of tumors with two different clinically relevant phenotypes.

Although nuclear medicine finds itself in exciting times as it advances the noninvasive whole-body assessment of cancer-specific physiologic and pathologic processes in patients, a major challenge exists. How to evaluate multiple phenotypes since PET cannot discriminate for positron emissions from different radiotracers, and how to achieve this in defined sequential manner that aims for the shortest possible time between first injection and final scan? Preclinical studies have previously utilized sequential injection-PET imaging to maximize diagnostic power for their particular investigations [33–35]. However, these studies still exercised caution by intentionally placing at least 1 day apart between the subsequent radiotracer injection and PET scan. Moreover, the long-circulating half-lives of antibodies further complicates a combination tracer approach to be achievable in an acceptable time window.

For example, Fowler et al. designed a study to determine whether the sequential injection of the radiotracers FES and [^18^F]fluorofuranylnorprogesterone specific for ER and the progesterone receptor (PR), respectively, could distinguish endocrine therapy-sensitive from resistant tumors [33]. Henry et al. evaluated [^89^Zr]Zr-DFO-transferrin for triple negative breast cancer and performed a head-to-head comparison with FDG [34]. Although sequential radiotracer injection and imaging was performed, it was with a 48-h delay between each tracer injection-PET session.

The key aim for this study was to demonstrate that in vivo targeting data for ^89^Zr-T was not compromised with respect to tumor contrast image quality. For clinical relevancy, it was particularly important to shorten the time between injection and scan for ^89^Zr-T due to its long biological half-life. There are numerous examples in the literature for preclinical imaging and biodistribution for ^89^Zr-T that match the data generated in this study. The data presented with ^89^Zr-T provide similarities and differences with previously published work. Janjigian et al. evaluated the targeting ability of ^89^Zr-T against HER2+ gastric cancer N87 tumors [36]. N87 has approximately the same number of HER2 receptors (~ 620–660 × 10^3^) on the cell surface as JIMT-1 cells (~ 4 × 10^5^ binding sites/cell) [37, 38]. When 8.1–10.2 MBq of ^89^Zr-T was administered 1 day following FDG-PET, the ROI-based tumor uptake values at 48 h p.i. were 20.0 ± 2.1 %IA/g and 7.4 ± 1.4 %IA/g for the HER2+ N87 and HER2− MKN-74 tumors, respectively. This is consistent with the uptake values of 16.2 ± 2.6 %IA/g and 6.8 ± 1.2 %IA/g in the JIMT-1 and MCF-7 tumors, respectively (Fig. 3b–d). Importantly, the T/negative tumor ratios were also similar (2.7 versus 2.4). This supports that injection of ^89^Zr-T immediately following 4FMFES-PET does not interfere with its ability to accurately visualize and quantify HER2+ tumors. Holland et al. administered ^89^Zr-T as a single imaging agent in female nude mice bearing HER2+ BT-474 and/or HER2− MDA-MB-468 xenografts [30]. At 48 h p.i., there was a T/M ratio of 7.9. In this study, ^89^Zr-T produced a T/M ratio of 6.7. As BT-474 expresses high levels of HER2, this would explain why the previous study has a slightly increased T/M ratio.

There were slight differences observed in ^89^Zr-T tumor uptake between the subcutaneous and orthotopic models. Two observations can be drawn from these results. Firstly, tumor uptake may be influenced by the implantation site, meaning that tumor microenvironment could be a significant factor when designing a dual tracer approach. Secondly, the presence of two tumors in the same mouse, with one lesion not expressing the tracer’s target, could act as a non-specific tumor sink. However, the latter explanation is most likely not occurring as ^89^Zr-T had higher uptake in JIMT-1 tumors in the heterotopic dual tumor-bearing model than in the orthotopic single tumor-bearing model. We therefore suggest that orthotopic and heterotopic models both be performed for future studies.

This study also utilizes ROI analysis in combination with biodistribution, which is a common practice in preclinical tracer development. As we relied solely on ROI analyses in the dual-tracer portion of this study, we had to ensure that the ROI analysis ^89^Zr-T, because it is the long circulating tracer, accurately reflected tumor, and non-specific tissue uptake as determined by the “gold-standard” biodistribution. Indeed, we demonstrated that in most cases for tumor and non-specific tissue uptake and T/M and T/B ratios ROI adequately reflected the biodistribution data. Nonetheless, there were discrepancies between bone uptake at late time points. The bone is difficult as the knee joint typically has increased uptake as it has more mass relative to cortical bone of the femur shaft. Hence, with ^89^Zr-labeled radiopharmaceuticals, this is an important anatomical site to consider.

The biological (whole-body residency time) half-life of ^89^Zr-T is a key factor for determining the optimal post-injection time and injected activity and the resulting dosimetry. Our studies in nude mice bearing HER2+ and HER2− tumors on opposite shoulders resulted in an elimination half-life of 234.4 h (Fig. 4a). Similarly, Holland et al. demonstrated that ^89^Zr-T in nude mice bearing single HER2+ BT-474 or HER2− MDA-MD-468 tumors had an elimination *t*_1/2_ of 150.5 ± 49.5 h and 336.2 ± 184.5 h, respectively [30]. The biological half-life of ^89^Zr-T in humans has been reported to be 111 h [39].

As such, the long half-life of ^89^Zr combined with the long residency time of trastuzumab means that dosimetry of ^89^Zr-T, and even more if combined with FDG or 4FMFES, might be a concern for routine clinical uses.

Serial imaging of JIMT-1 tumor-bearing mice showed a substantial gain in tumor contrast between 24 h and 48 h p.i., which doubled again between 72 h and 144 h. While images of adequate quality can be obtained as soon as 48 h p.i., optimal detection of HER2+ tumors necessitates later time imaging, which might require increased injected activities to not compromise image quality.

Human dosimetry for FDG, 4FMFES, and ^89^Zr-T was already reported, and the significant contributor is ^89^Zr-T with an average effective dose of 48.6 mSv [40], which is ~ 10-fold higher than the typical mean effective dose of either FDG (5.6 mSv) [41] or 4FMFES (4.8 mSv) [42] PET scans. Hence, the use of both 4FMFES and ^89^Zr-T will only increase the effective dose by ~ 10% compared to ^89^Zr-T-PET scan alone, which could be acceptable if the clinical benefits of the additional information from the second tracer more than compensate for the increased radiation exposure compared to its individual components. Such a risk-to-benefit ratio assessment will need to be further investigated in the future.

Thus, the prolonged residency half-life of ^89^Zr-T reported in preclinical models, including this study, and in humans justifies further refinement to shorten blood clearance and elimination. Future studies will incorporate radiolabeled F(ab′)_2_ or engineered antibody fragments of HER2-targeting monoclonal antibodies in order to shorten the injection-to-target time and decrease radiation dose. Such innovations will further facilitate the implementation of this dual-tracer approach for improved PET-guided whole-body detection and phenotyping of breast cancers.

## Conclusion

In conclusion, this study developed a sequential injection-imaging protocol for 4FMFES-PET followed by ^89^Zr-T-PET and determined it is feasible to obtain high contrast specifically targeted images of the ER+/HER2− MCF-7 and ER−/HER2+ JIMT-1 tumors. This study provides a first of its kind protocol combined with fast and slow-clearing tracers with the intentional aim to image two clinically relevant phenotypes in an acceptable time window and provides the preclinical conditions to develop an anti-ER/HER2 molecular imaging tool kit for potential clinical application.

## Supplementary information

**Additional file 1.** a) Autoradiography of a typical SDS-PAGE in non-reducing condition of a 1 μg sample of ^89^Zr-T. The presence of a single band at 150 kDa shows the absence of higher molecular weight aggregates and of lower molecular weight fragments. b) Autoradiography of a typical thin-layer chromatograph of 1 kBq ^89^Zr-T (left lane) and of 0.3 kBq unchelated ^89^Zr-oxalate at pH 7.0 (right lane). Elution was achieved with a 0.1 M DTPA solution. Radiopurity of ^89^Zr-T was over 95% in all experiments in this study.

## Data Availability

The datasets used and/or analyzed during the current study are available from the corresponding author on reasonable request.

## Abbreviations

^89^Zr-T: [^89^Zr]Zr-DFO-trastuzumab
4FMFES: 4-Fluoro-11β-methoxy-16α-[^18^F]fluoroestradiol
%IA/g: Percent of injected activity per gram of tissue
ANOVA: Analysis of variance
ATCC: American Type Culture Collection
DFO: Deferoxamine
DMEM: Dulbelco’s Modified Eagle Medium
ER: Estrogen receptor
FDG: 2-Deoxy-2-[^18^F]fluoro-D-glucose
FES: 16α-[^18^F]fluoroestradiol
HER2: Human epidermal growth factor receptor 2
IHC: Immunohistochemistry
PET: Positron emission tomography
p.i.: Post-injection
ROI: Region of interest
SDS-PAGE: Sodium dodecyl sulfate polyacrylamide gel electrophoresis
T/M: Tumor-to-muscle
