# Supplementary figures and images for: A preclinical PET dual-tracer imaging protocol for ER and HER2 phenotyping in breast cancer xenografts
Source: EJNMMI Res. 2020 Jul 3;10:69. doi: 10.1186/s13550-020-00656-8 (PMC7334319; doi:10.1186/s13550-020-00656-8)

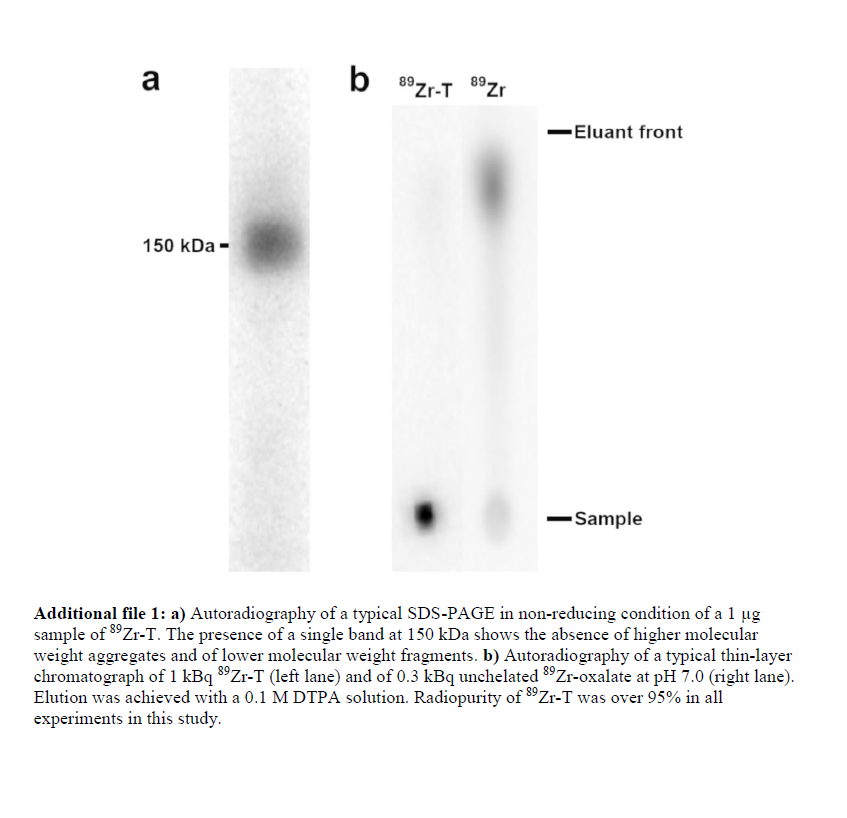

Supplement: Supplementary file 1 — Additional file 1. a) Autoradiography of a typical SDS-PAGE in non-reducing condition of a 1 μg sample of 89Zr-T. The presence of a single band at 150 kDa shows the absence of higher molecular weight aggregates and of lower molecular weight fragments. b) Autoradiography of a typical thin-layer chromatograph of 1 kBq 89Zr-T (left lane) and of 0.3 kBq unchelated 89Zr-oxalate at pH 7.0 (right lane). Elution was achieved with a 0.1 M DTPA solution. Radiopurity of 89Zr-T was over 95% in all experiments in this study. [file 13550_2020_656_MOESM1_ESM.png]
